# Targeting VDR–RXR heterodimerization in neurodegenerative diseases: a hypothetical framework for combined vitamin D_3_ and vitamin A therapy

**DOI:** 10.3389/fneur.2026.1754364

**Published:** 2026-04-10

**Authors:** Mujittapha Umar Sirajo, Rukevwe Obie, Abubakar I. Mukhtar, Nasiru M. Abdullahi, Enaohwo M. Taniyohwo, John C. Oyem, Ibrahim M. Badamasi

**Affiliations:** 1 Department of Anatomy, Federal University of Health Science, Azare, Nigeria; 2 ARUA Center of Excellence for Non-Communicable Diseases, University of Nairobi, Nairobi, Kenya; 3 Department of Anatomy, Delta State University, Abraka, Nigeria; 4 Department of Anatomy, Ahmadu Bello University, Zaria, Nigeria; 5 Department of Community, Psychiatric and Mental Health, College of Nursing, Qassim University, Buraydah, Saudi Arabia; 6 Department of Nursing, Medical City, Qassim University, Buraydah, Saudi Arabia; 7 Department of Anatomy, Novena University, Ogume, Nigeria; 8 Anatomy Unit, Faculty of Basic Clinical Sciences, The University of the West Indies at St Augustine, Saint Augustine, Trinidad and Tobago

**Keywords:** Alzheimer’s disease, Parkinson’s disease, neuroprotection, VDR–RXR heterodimer, vitamin A, vitamin D3

## Abstract

Neurodegenerative diseases such as Alzheimer’s and Parkinson’s disease are characterized by progressive neuronal loss, oxidative stress, and limited treatment options. While vitamin D₃ has demonstrated neuroprotective potential, we hypothesize that its co-administration with vitamin A may enhance therapeutic effects via synergistic interactions between their nuclear receptors (the vitamin D Receptor (VDR) and Retinoid X Receptor (RXR)). The interaction leads to the formation of a heterodimer, which regulates genes involved in neuronal survival, inflammation, and oxidative balance. A comprehensive literature review was conducted to evaluate the mechanisms underlying Vitamin D₃'s neuroprotection and Vitamin A’s modulatory role through RXR activation, focusing on studies exploring the VDR-RXR heterodimer in Alzheimer’s and Parkinson’s disease models. Evidence indicates that vitamin D₃ mitigates neurodegeneration by upregulating neuroprotective genes, reducing oxidative stress, and modulating calcium homeostasis, with these effects amplified by RXR activation. The VDR-RXR heterodimer interaction appears critical for enhancing transcriptional activity, promoting neuronal resilience, while potentially slowing neurodegeneration progression. We propose that combined vitamin D₃ and vitamin A supplementation could offer a promising therapeutic strategy by synergistically optimizing VDR-RXR signaling, thereby improving neuroprotection. This hypothesis requires validation through an integrated approach that includes molecular, cellular, behavioral, and translational neuroimaging methods to investigate neuroprotective effects associated with VDR-RXR co-activation.

## Introduction

Neurodegenerative diseases such as Alzheimer’s disease (AD) and Parkinson’s disease (PD) are characterized by progressive neuronal dysfunction and cell death, leading to cognitive and motor impairments (1). Despite decades of research, available treatments largely address symptoms without effectively halting or reversing disease progression (2). Increasing attention is directed toward nutritional interventions, particularly the role of vitamins with antioxidant and neuroprotective properties as adjunct strategies to mitigate neurodegeneration (3). Among these, vitamin D₃ has emerged as a key modulator of neuronal survival, inflammation, and calcium homeostasis. Because VDR heterodimerization with RXR is required for full therapeutic potential, any deficiency or suboptimal activation of RXR may affect vitamin D3’s effect. Recognizing this dependency clarifies the biological rationale for investigating combined vitamin D3 and vitamin A supplementation as a potential synergistic neuroprotective strategy (4).

**Figure 1:**
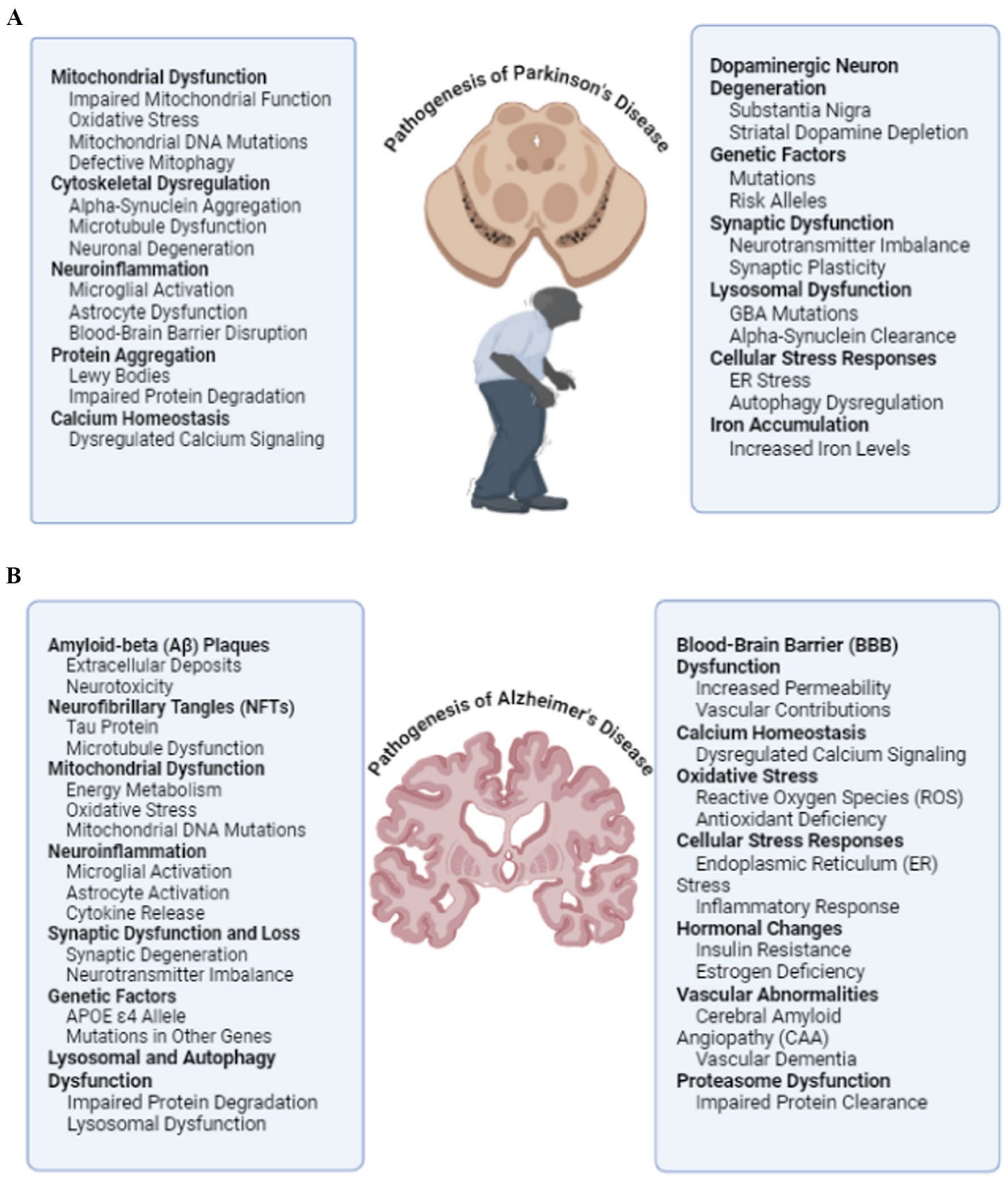
**(A)** Contributing factors to pathogenesis of Parkinson’s disease, leading to the characteristic motor and non-motor symptoms of the disorder. **(B)** Contributing factors to progressive neurodegeneration and cognitive decline in Alzheimer’s disease.

Recent studies have identified several converging pathogenic mechanisms that contribute to the onset and progression of neurodegenerative diseases (NDs) (5, 6). These include excitotoxicity, dysregulation of calcium homeostasis, mitochondrial dysfunction, activation of proteolytic enzymes, excessive production of reactive oxygen species (ROS), and a compromised antioxidant defense system (Figure 1A) (7–9). Such disturbances lead to neuronal damage and apoptosis, contributing significantly to neurodegeneration. In addition, chronic neuroinflammation, cytoskeletal abnormalities, and deficiencies in neurotrophic support further exacerbate neuronal vulnerability and loss (10–13). In AD, pathological features such as extracellular accumulation of amyloid-beta (Aβ) plaques and intracellular neurofibrillary tangles disrupt synaptic function and trigger neuronal death. While in PD, the pathologic hallmarks include progressive degeneration of dopaminergic neurons in the nigrostriatal pathway and the formation of intracellular α-synuclein aggregrates known as Lewy bodies (14, 15). Despite significant advances in understanding PD and AD, the multifactorial and interconnected nature of these mechanisms continues to hinder the development of effective, disease-modifying therapies, leaving most current treatments symptomatic and insufficient to prevent or reverse disease progression.

**Figure 2:**
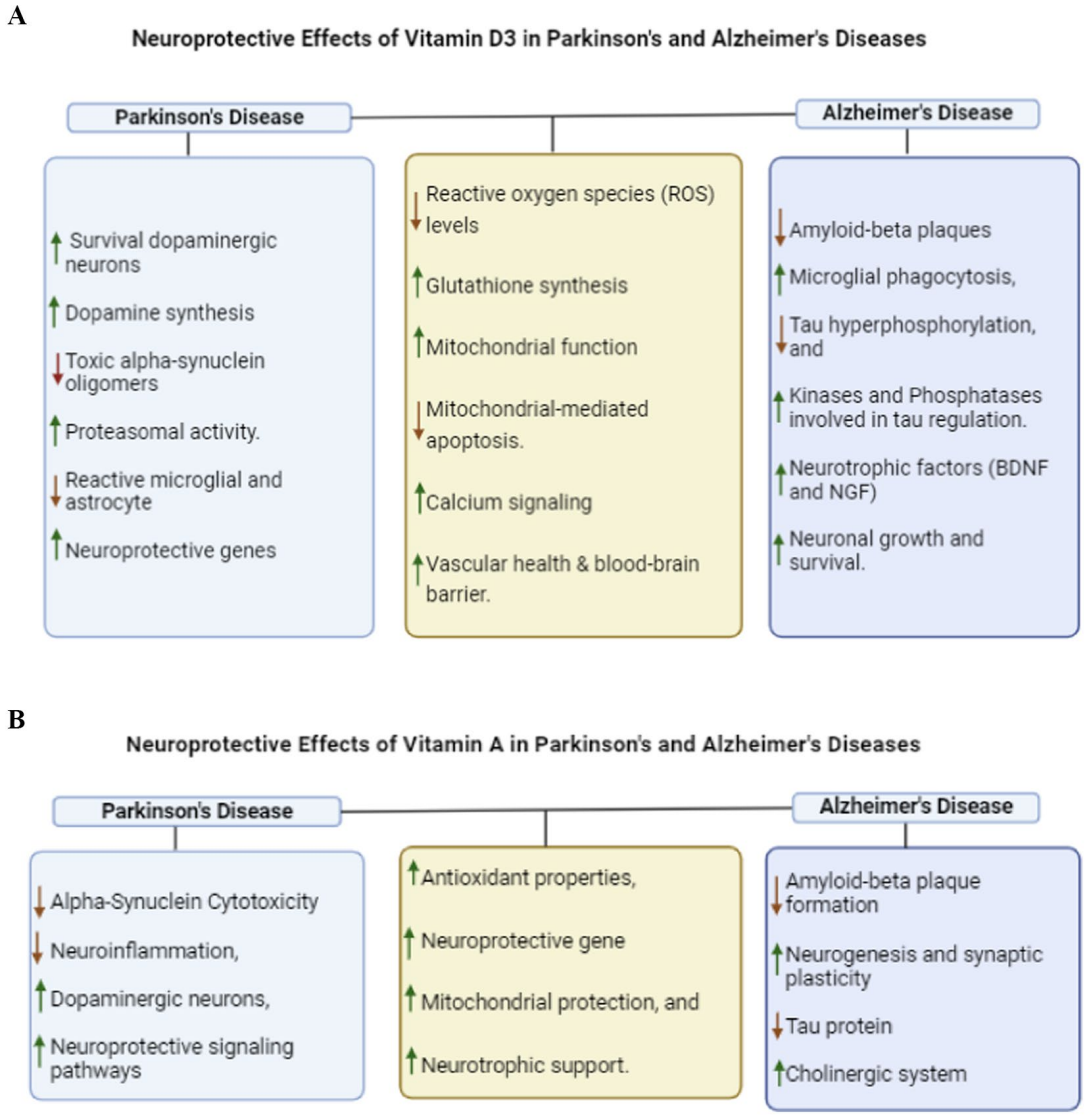
**(A)** Neuroprotective effect of vitamin D_3_ in Parkinson’s disease and Alzheimer’s diseases in preclinical studies. **(B)** Neuroprotective effect of vitamin A in Parkinson’s disease and Alzheimer’s diseases in preclinical studies.

Moreover, the limited effectiveness of many neuroprotective agents in preclinical studies has driven ongoing efforts to identify multi-targeted compounds with robust therapeutic potential. Notably, numerous preclinical studies have highlighted the promising neuroprotective effects of vitamin D₃ administration in rodent models of neurodegenerative diseases (NDs) (16–21). Our laboratory, along with others, has demonstrated that activation of the vitamin D Receptor (VDR) alone can significantly improve motor and cognitive impairments (9, 21–24) and promote neuronal survival by enhancing cellular repair mechanisms. (25–27) VDR activation plays a central role in upregulating genes involved in protein synthesis, mitigating calcium-induced cytotoxicity, and reducing reactive oxygen species (ROS) production—key factors implicated in the progression of neurodegeneration (Figure 2A) (28–31). Given significant roles in the striatum, targeting VDR represents a promising strategy to modulate pathological changes observed in AD and PD. (32–35)

In the same vein, emerging evidence has demonstrated that activation of the Retinoid X Receptor (RXR) via Vitamin A also confers significant neuroprotective effects through mechanisms that parallel those of Vitamin D₃ (36). Several active metabolites of vitamin A, particularly retinoic acid, have been shown to play pivotal roles in neuronal differentiation, neurogenesis, and synaptic plasticity (Figure 2B), thereby supporting both cognitive and motor functions (37). In addition to modulating gene expression, retinoic acid exhibits potent antioxidant and anti-inflammatory properties, which contribute to the attenuation of oxidative stress and chronic neuroinflammation in the central nervous system. In models of AD, PD, and amyotrophic lateral sclerosis (ALS), Vitamin A derivatives were reported to reduce amyloid-*β* plaque accumulation and protect against dopaminergic neuronal degeneration associated with oxidative insults (38). Collectively, these findings highlight the therapeutic potential of retinoic acid as a multifaceted neuroprotective agent. However, despite encouraging preclinical results, further clinical investigations are required to validate its efficacy and determine optimal dosing regimens for translational application.

**Figure 3:**
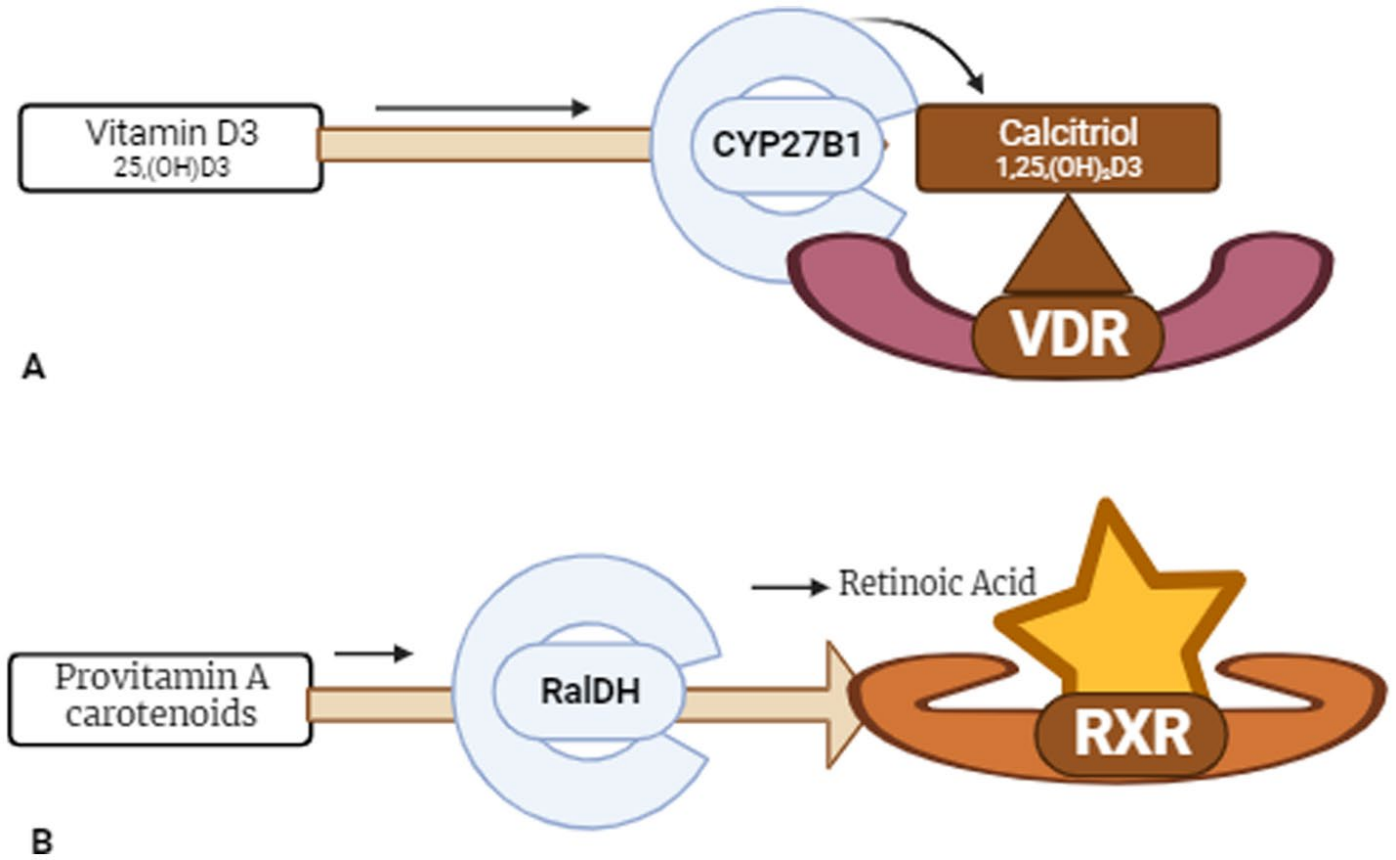
Schematic illustration of the conversion from inactive forms of vitamin D and vitamin A to their active forms and their binding to respective receptor sites. **(A)** Depicts the conversion of the inactive form of vitamin D, cholecalciferol or vitamin D_3_ [25(OH)D_3_/25-hydroxyvitamin D], into its active form, calcitriol, facilitated by the enzyme CYP27B1. The active form subsequently binds to its receptive site, VDR (vitamin D receptor). **(B)** Illustrates the transformation of the inactive form of vitamin A, provitamin A carotenoids, into the active form, retinoic acid, through the action of Retinaldehyde Dehydrogenase (RalDH). The active form subsequently binds to its receptive site, RXR (retinoid X receptor).

**Figure 4:**
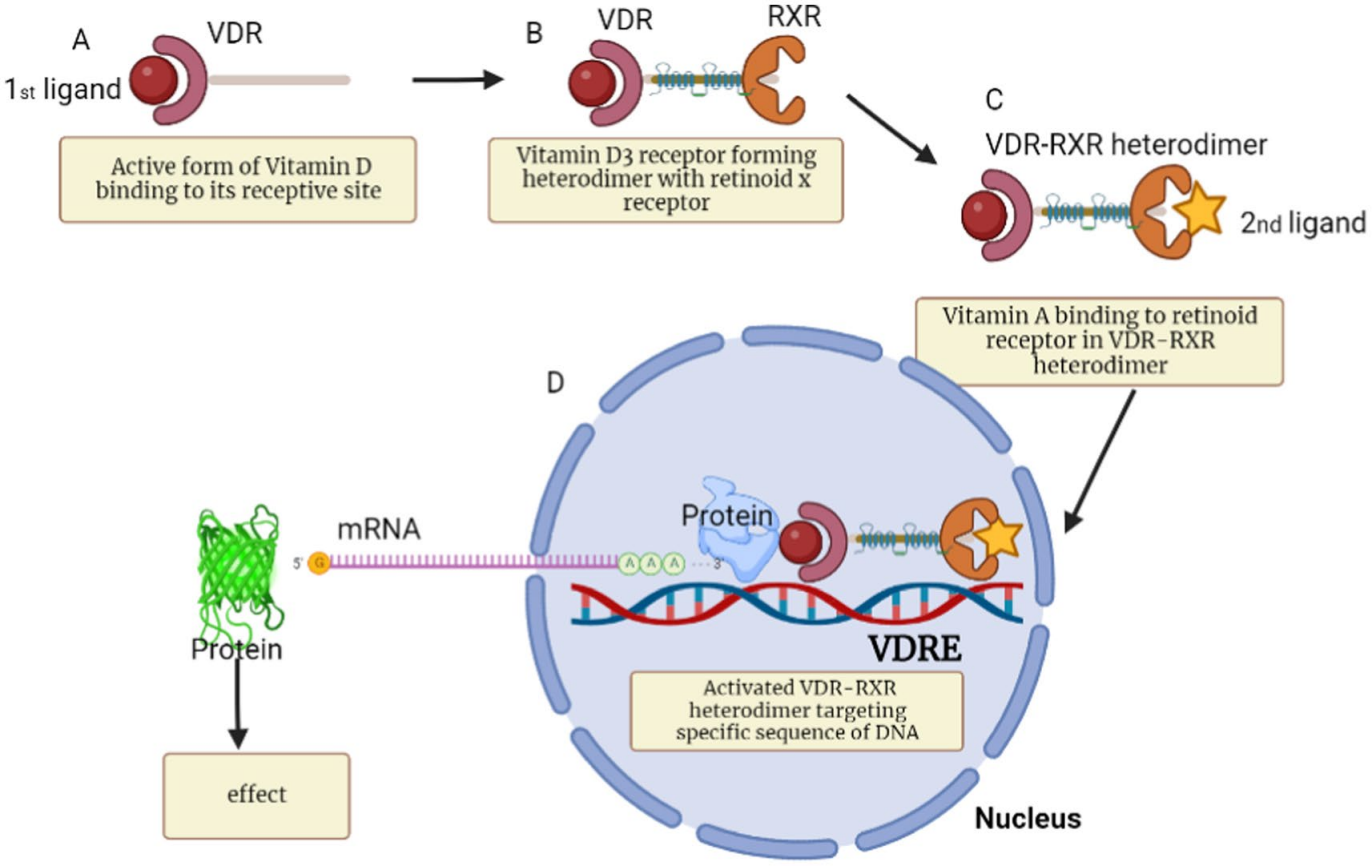
Illustrative description of the complex process involved in the biological activities of vitamin D_3_ receptor and its interaction with retinoid X receptor to form VDR-RXR heterodimer. **(A)** Shows how a ligand binds to vitamin D receptor. **(B)** Shows how ligand activated VDR interacting with RXR to form VDR-RXR heterodimer. **(C)** Show how the second ligand bind to retinoid X receptor that is located in the VDR-RXR heterodimer complex. **(D)** Shows how the heterodimer complex bind to specific DNA sequence (VDRE) leading to the recruitment of other proteins such as RNA polymerase, which then transcribe the DNA and produce mRNA that is related to vitamin D. The mRNA subsequently leaves the nucleus and is transported into the cytoplasm where it is translated in to protein that are responsible for some biological effects such as anti-inflammation and others highlighted in Figure 2A.

The neuroprotective efficacy of vitamin D₃ is linked to its ability to form a functional heterodimer with the Retinoid X Receptor (RXR), a process that significantly amplifies its regulatory potential. (38–41) The biologically active form of vitamin D₃ binds to the VDR, which subsequently heterodimerizes with RXR, each receptor being activated by its respective ligand (Figures 3, 4) (42, 43). This VDR–RXR complex is essential for mediating gene transcriptions involved in neuronal survival, anti-inflammatory responses, and antioxidant defenses (44). We hypothesized that administering vitamin D₃ alone, without concurrent RXR activation by Vitamin A, may lead to suboptimal VDR–RXR activation and thus attenuate the full neuroprotective potential of vitamin D₃. This incomplete receptor engagement could compromise therapeutic efficacy in the context of neurodegenerative diseases. Therefore, understanding and leveraging the synergistic interaction between VDR and RXR presents a promising frontier for developing more effective, receptor-targeted interventions in AD, PD, and related neurodegenerative conditions. Accordingly, this article presents a mechanistic hypothesis and outlines specific preclinical and clinical experimental strategies to evaluate whether dual activation of VDR and RXR enhances neuroprotection beyond vitamin D3 alone.

### Hypothesis

If vitamin D3, administered alone, activates the VDR but fails to concurrently activate the RXR, probably due to insufficient coupling with vitamin A, then the resulting VDR-RXR heterodimer complex may not attain maximum activation. This suboptimal activation could potentially limit the biological efficacy of vitamin D3. Therefore, we hypothesized that combined vitamins D3 and A supplementation could yield better neuroprotective outcomes in the treatment of neurodegenerative diseases by synergistically modulating the VDR-RXR complex.

If vitamin D₃ (vitamin D3) and vitamin A synergistically enhance vitamin D receptor-retinoid X receptor (VDR-RXR) activation, then molecular markers of antioxidant and neurotrophic signaling—such as superoxide dismutase (SOD) and brain-derived neurotrophic factor (BDNF)—will increase. Downstream imaging biomarkers, including reduced lesion burden on fluid-attenuated inversion recovery (FLAIR) sequences, improved fractional anisotropy on diffusion tensor imaging (DTI), preserved hippocampal subfields, and strengthened functional connectivity on functional magnetic resonance imaging (fMRI), will likely reflect these biological improvements.

### Evaluation of the hypothesis

The formation of a heterodimer complex between the VDR and the RXR is essential for the full transcriptional activity of vitamin D₃. RXR activation by retinoids has been shown to enhance the stability and transcriptional efficacy of this complex, indicating that co-activation of both receptors may be critical for optimal biological function. Notably, vitamin A, through its interaction with RXR, has independently been implicated in neuroprotective mechanisms. (44) Two different studies reported that vitamin A deficiency impairs RXR-mediated signaling, potentially attenuating the neuroprotective capacity of VDR-bound vitamin D₃ (45, 46). This interaction becomes particularly relevant in neurodegenerative disease conditions, where enhanced neuroprotection may be achieved through co-administration of vitamin D₃ and retinoids. Supporting this notion, Dursun et al. (47) demonstrated that the combined administration of vitamin D₃ and Vitamin A derivatives yielded significantly greater neuroprotective outcomes in models of neurodegeneration compared to vitamin D₃ monotherapy. Furthermore, a recent finding from our laboratory demonstrated that animals receiving co-administration of vitamin D₃ and vitamin A show enhanced neuronal survival and functional outcomes compared to those receiving Vitamin D₃ alone (48). These findings suggest that RXR activation via vitamin A may potentiate VDR-mediated signaling, thereby amplifying the therapeutic effects of vitamin D₃.

While substantial evidence indicates that vitamin D₃ exerts beneficial effects in neurodegenerative diseases when administered alone, the consistency and magnitude of these effects remain variable. Some clinical studies have reported improved cognitive or neurological outcomes following vitamin D supplementation (49), while others show inconsistent or modest benefits (50). This variability raises the possibility that vitamin D₃ may exert partial or suboptimal effects in the absence of concurrent RXR activation. Although endogenous dietary intake together with the retinoic acid receptors present in the brain may provide vitamin A to activate RXR under normal physiological conditions, there is insufficient evidence to conclude that such basal levels consistently support optimal VDR-RXR activity across diverse disease states (46). To establish whether VDR activation alone is sufficient for neuroprotection, different experimental approaches such as targeted RXR knockdown or genetic ablation would be necessary. Such studies could delineate the extent to which RXR contributes to VDR-mediated transcriptional regulation in the absence of exogenous Vitamin A. It is also important to mention that VDR-RXR signaling is regulated not only by ligand availability but also by a complex interplay of additional factors. These include the recruitment of nuclear coactivators, chromatin accessibility, and the organism’s metabolic state, all of which modulate the transcriptional efficacy of the VDR-RXR heterodimer (51). These elements collectively modulate the transcriptional activity of the VDR-RXR complex, adding layers of regulation that influence gene expression outcomes. For instance, in a relaxed chromatin state, the VDR-RXR complex can efficiently recruit coactivators and the basal transcriptional machinery, facilitating gene activation (52). However, this activation is tightly controlled and transient, as ligand metabolism, receptor degradation, and chromatin remodelling can rapidly alter the signaling dynamics (53).

Furthermore, the VDR-RXR complex is involved in both gene activation and repression, and the mechanisms governing these opposing outcomes are diverse and context-dependent. Therefore, while RXR activation may enhance the neuroprotective effects of vitamin D₃, it is not the sole determinant of VDR-RXR signaling efficacy. The overall biological response is shaped by the integration of ligand presence, coactivator availability, chromatin state, and broader cellular context, underscoring the complexity of targeting this pathway for therapeutic benefit (54). Taken together, our hypothesis that vitamin A enhances the therapeutic efficacy of vitamin D₃ via RXR activation is supported by preclinical studies demonstrating improved receptor complex formation and increased neuroprotection with combined treatment (55). However, it is important to note that VDR possesses intrinsic activity even in the absence of RXR activation, and nuclear receptor signaling is inherently multifactorial. This suggests that while vitamin A is a significant modulator of vitamin D₃-mediated neuroprotection, it is not strictly indispensable for this effect. Future research, particularly well-controlled clinical trials directly comparing isolated and combined supplementation will be essential to fully elucidate the translational potential and therapeutic relevance of these interactions.

### Hypothesis testing and implication

The current hypothesis raised by this paper can be explored through both basic and clinical research approaches aimed at elucidating the combined role of VDR and RXR in mitigating AD and PD. A comprehensive evaluation of this hypothesis has the potential to revolutionize current paradigms in neurodegenerative disease research and treatment.

#### Basic research

One fundamental approach for testing this hypothesis is basic preclinical research. Establishing an *in vivo* animal model or an *ex vivo* model of neurodegenerative disease is an initial step in the research process. The models mimic the disease phenotypes of PD and AD, using a valid approach. In PD, neurotoxins like MPTP (1-methyl-4-phenyl-1,2,3,6-tetrahydropyridine) and 6-OHDA (6- hydroxydopamine) are commonly used. (52) MPTP and 6-OHDA are known agents that selectively induce dopaminergic neuronal loss, mirroring PD-like characteristic degeneration of dopaminergic cells with associated motor deficits (56).

**Figure 5:**
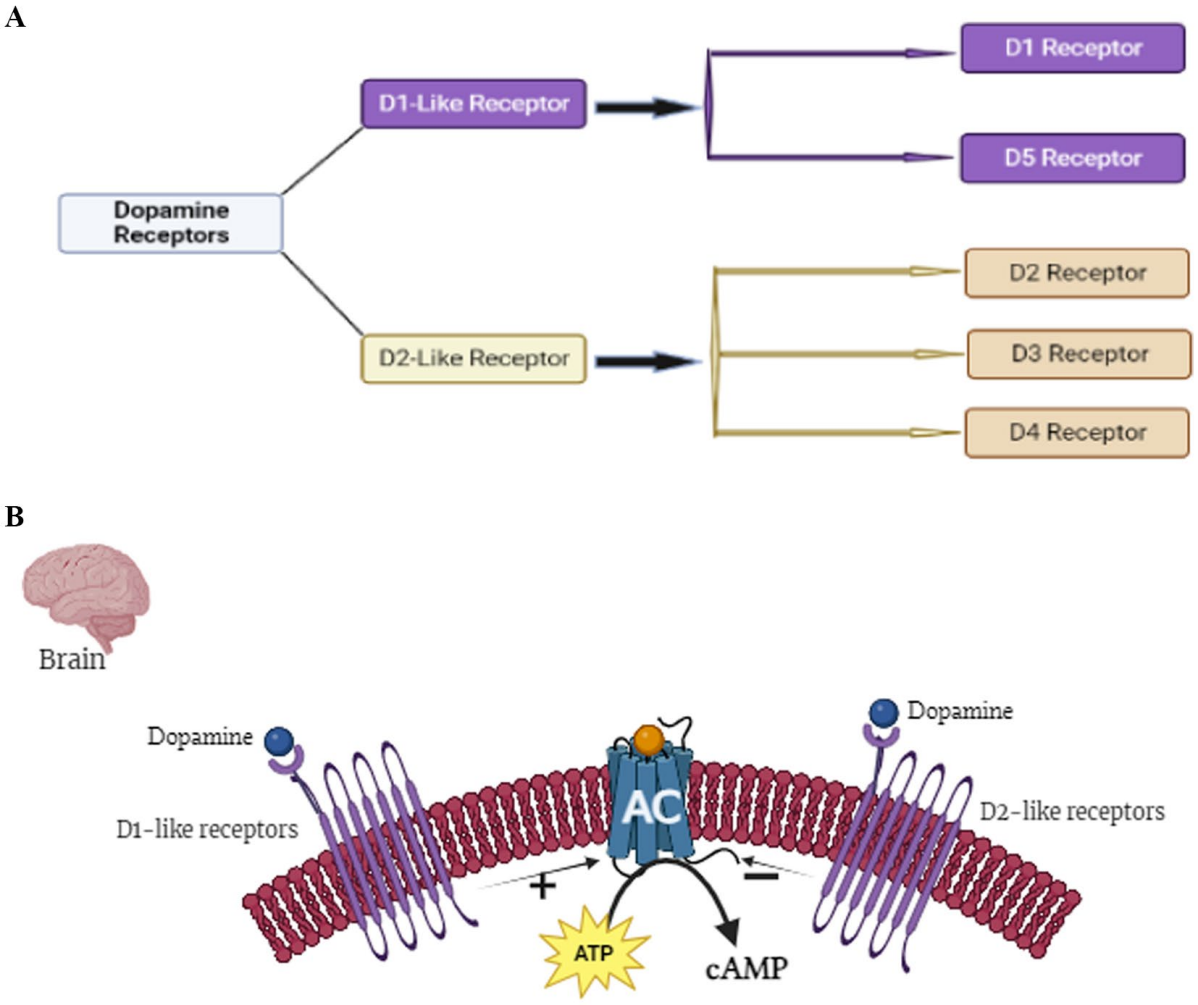
**(A)** Classification of dopamine receptor. There are five dopamine receptors: D1, D2, D3, D4, and D5. These receptors are classified as D1-like receptor or D2-like receptors. D1 and D5 are regarded as D-like receptors, whereas D2, D3, and D4 are regarded as D2-like receptor. **(B)** Illustration of the regulation of adenylyl cyclase (AC) by D1-like and D2-like receptors in the brain. The figure visually represents the modulation of adenylyl cyclase activity in response to dopamine signaling. Specifically: The illustration demonstrates the binding of dopamine molecules to D1-like receptors, resulting in the stimulation of adenylyl cyclase activity. Conversely, the figure illustrates that when dopamine binds to D2-like receptors, this interaction inhibits adenylyl cyclase.

Additionally, D2 receptor blockers, such as haloperidol, can be employed to induce Parkinsonism- like symptoms, and they act by disrupting dopaminergic signaling (Figure 5) (54, 55). AD models, on the other hand, have commonly been developed through genetic manipulation to overexpress mutant forms of proteins such as beta-amyloid (Aβ) or tau (57). Alternatively, Aβ aggregates may be directly injected into the brain of animals to replicate the hallmark amyloid plaques observed in AD (57). In addition to genetic and peptide-based models, several neurotoxins, such as streptozotocin (STZ), colchicine, and scopolamine, have been employed to induce Alzheimer-like phenotypes by promoting oxidative stress, neuroinflammation, cholinergic deficits, and cognitive impairment, thereby mimicking various pathological features of the AD (58).

The grouping for this hypothesis testing should include distinct categories to assess the effects of each treatment modality systematically. Firstly, there should be either an AD or PD model group that will serve as the baseline for comparison with other groups (positive control). Then, there should be an RXR Group receiving treatment aimed at stimulating RXR, allowing for the assessment of its individual effects. A VDR Group would receive treatment targeting VDR alone, enabling the evaluation of VDR activation independent of RXR. Finally, a VDR-RXR group would receive combined treatment targeting both receptors, facilitating the investigation of potential synergistic effects.

To further elucidate the mechanisms underlying the synergistic neuroprotective effects of vitamin A and vitamin D₃ via RXR and VDR activation, follow-up experiments utilizing genetic knockout models would be highly informative. Specifically, targeted deletion (knockout) of the vitamin D receptor (VDR) or the retinoid X receptor (RXR) in neuronal cell lines or animal models can help dissect the individual and combined contributions of these receptors to neuroprotection. For example, VDR knockout mice have already been developed and characterized, revealing significant alterations in gene transcription, behavior, and neuroprotection, largely due to the loss of VDR-mediated signaling (59). Similarly, RXR knockout models can be used to evaluate the impact of RXR deficiency on neuronal survival, stress response, and inflammation, as RXR ligands have demonstrated neuroprotective effects in various neurodegenerative disease models (60).

By conducting experiments in which either VDR or RXR is knocked out—using technologies such as CRISPR-Cas9 or Cre-Lox P recombination, researchers can administer vitamin D₃, vitamin A, or their combination and assess outcomes including neuronal viability, synaptic function, oxidative stress markers, and neuroinflammatory responses (61). Comparing these results with wild-type controls will clarify whether the neuroprotective effects are dependent on the presence of both receptors or can occur independently through alternative pathways. Additionally, these knockout studies can be complemented by transcriptomic and proteomic analyses to identify downstream targets and signaling networks uniquely regulated by VDR, RXR, or their heterodimeric complexes (61, 62). This approach will provide mechanistic insights into how vitamin A and vitamin D₃ interact at the molecular level to mediate neuroprotection and may help identify novel therapeutic targets for neurodegenerative diseases. Experiments employing VDR and RXR knockout models represent a powerful strategy to unravel the receptor-specific mechanisms driving vitamin-mediated neuroprotection, ultimately guiding the development of more effective combinatorial therapies.

Assessing the functional outcomes of molecular and pharmacological manipulations in a battery of neurobehavioral tests is essential for evaluating both motor and cognitive domains relevant to PD and AD (63–67). In PD models, motor deficits can be assessed using tests such as the rotarod performance test, which evaluates balance and coordination, and the open field test, which measures general locomotor activity and anxiety-related behavior (68, 69). The cylinder test and pole test are also commonly employed to evaluate forelimb use asymmetry and bradykinesia, respectively. For AD models, cognitive impairment is typically assessed using spatial memory and learning paradigms, such as the Morris water maze and Y-maze, which evaluate hippocampal-dependent spatial navigation and working memory (70, 71). Novel object recognition (NOR) tests assess recognition memory, while passive avoidance and fear conditioning paradigms measure associative memory and learning. These behavioral assessments provide crucial endpoints to correlate with molecular and imaging data, facilitating comprehensive evaluation of the impact of VDR and RXR modulation on neurodegenerative phenotypes (72–76). Standardizing and integrating these tests across experimental groups ensures robust evaluation of motor and cognitive functions, thereby validating the therapeutic potential of co-administering vitamin D3 and vitamin A in PD and AD models.

To investigate the impact of the VDR and RXR in AD and PD models, a comprehensive approach involving multiple parameters and methodologies is essential. Molecular biology techniques, including western blotting and immunoprecipitation, could be vital for assessing protein expression levels associated with RXR and VDR. It is imperative to standardize the expression of proteins in each experimental setting using housekeeping proteins such as GAPDH (Glyceraldehyde-3-phosphate dehydrogenase) and other commonly used internal controls in western blotting (77). Additionally, gene expression analyses using qRT-PCR to quantify mRNA levels of VDR and RXR in response to the varying experimental conditions for the different groups should be conducted as follow-up experiments (78). Furthermore, imaging techniques such as fluorescence microscopy or confocal imaging can be employed to visualize the subcellular localization and co-localization of VDR and RXR within nerve cells. To determine whether RXR stimulation is necessary for VDR function, pharmacological inhibitors or genetic knockdown/knockout approaches can selectively block RXR activity and assess its impact on VDR-mediated signaling pathways. Additionally, functional assays, such as luciferase reporter assays, should be conducted to measure VDR transcriptional activity in the presence or absence of RXR stimulation (79). To sum up, employing these comprehensive experimental strategies, including behavioral studies, will elucidate the intricate relationship between VDR and RXR and determine whether RXR stimulation is necessary for optimal VDR function.

Subsequently, to study the interactions between Vitamin A and Vitamin D3 *in vitro,* researchers can employ receptor-binding assays, reporter-gene assays, chromatin immunoprecipitation (ChIP) assays, electrophoretic mobility shift assays (EMSA), and co-immunoprecipitation (Co-IP) assays (80). These methods will enable detailed characterization of the binding affinities, transcriptional activities, DNA-binding dynamics, and physical interactions of VDR and RXR receptors in the presence of these vitamins. Expected outcomes may include demonstration of enhanced VDR-RXR heterodimer formation and transcriptional synergy (e.g., 2-3-fold upregulation of vitamin D response element-driven luciferase activity), identification of key coactivator recruitment sites via ChIP-seq peaks, and confirmation of dose-dependent binding shifts via EMSA band intensities. Such results would provide mechanistic evidence supporting the s neuroprotective potential of combined vitamin A and D₃ therapy over vitamin D₃ alone, paving the way for *in vivo* validation in rodent models of PD and AD. Altogether, transcriptomics, proteomics, and metabolomics approaches provide comprehensive insights into the combined nutritional impact and ADME (Absorption, Distribution, Metabolism, and Excretion) profiles of vitamin A and D3 (40). These omics studies will facilitate the identification of gene expression changes, protein modifications, and metabolic pathway alterations, aiding in elucidating the synergistic or antagonistic effects of these vitamins before *in vivo* studies.

**Figure 6:**
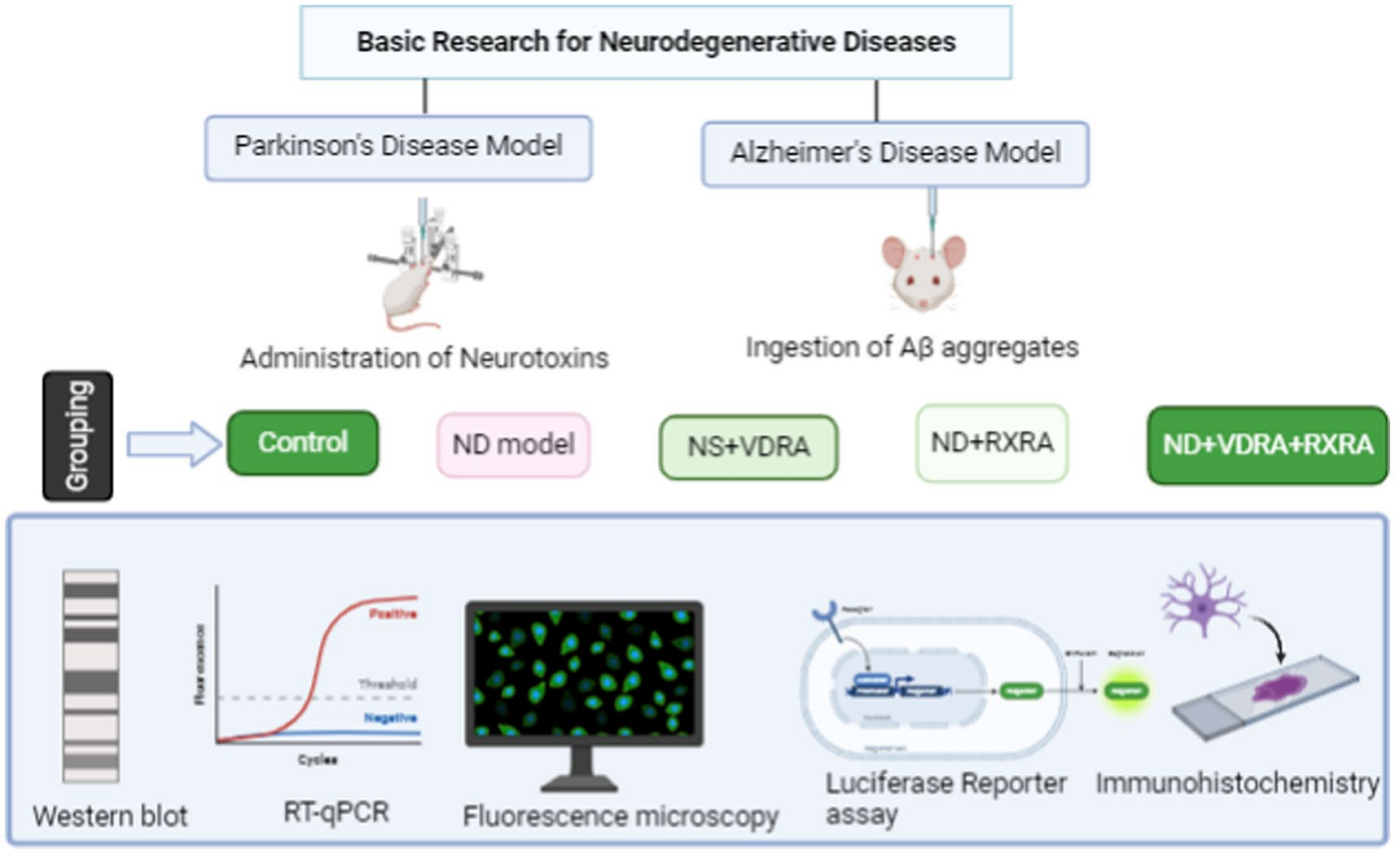
Illustrative diagram of preclinical studies investigating the role of VDR and RXR in neurodegenerative diseases. This figure depicts the comprehensive preclinical research approach to testing the hypothesis involving vitamin D_3_ receptor (VDR) and retinoid X receptor (RXR) in Parkinson’s disease (PD) and Alzheimer’s disease (AD).

Another research approach will be to incorporate preclinical neuroimaging techniques, which will provide non-invasive markers of treatment response. With FLAIR, for example, researchers quantify white matter hyperintensities and lesion-like changes associated with oxidative stress, inflammation, and vascular injury. Diffusion tensor imaging DTI offers a metric of microstructural integrity [e.g., fractional anisotropy (FA), linking connectivity changes to behaviors or conditions (40, 58)]. fMRI, on the other hand, examines brain function and activity patterns in response to stimuli or interventions, likely uncovering neural circuit involvement in various processes (81). Using these approaches across different experimental groups could provide detailed insights into underlying mechanisms, such as alterations in white matter integrity or neural network responses, thereby enhancing our understanding of brain function in both healthy and pathological states (see Figure 6 for an illustrative preclinical research approach to testing the hypothesis).

#### Clinical research

In clinical research, neuroimaging techniques such as magnetic resonance imaging (MRI) and positron emission tomography (PET) provide powerful tools for investigating the roles of nuclear receptors such as VDR and RXR in neurodegenerative diseases. Structural MRI provides high- resolution anatomical data that enables the detection of brain atrophy patterns characteristic of AD and PD. These atrophy patterns, such as hippocampal and cortical thinning in AD, or specific regional atrophy in PD, are established markers of neurodegeneration and correlate with clinical symptoms and disease progression. Since structural MRI is widely used for diagnosis, monitoring, and even predicting the course of these diseases, it will provide readily available longitudinal biomarkers that will track neurodegeneration and correlate it with clinical decline in PD and AD.

While MRI-detected atrophy robustly indicates neuronal loss and disease severity in PD and AD, it does not directly measure disruptions in receptor-mediated neuroprotection (e.g., VDR-RXR signaling). Instead, atrophy indirectly reflects breakdowns in these pathways, as neuronal survival and synaptic integrity depend on such signaling to counter oxidative stress and neuroinflammation (82). FLAIR sequences detect associated white matter hyperintensities from small vessel injury, while DTI and fMRI assess microstructural damage and network reorganization, respectively, thus providing complementary, downstream readouts of neuroprotective efficacy. To date, no direct receptor imaging exists clinically, so this multi-modal cascade (FLAIR+DTI + fMRI) seems promising to test our hypothesis indirectly. Continuing in this train of thought, these multimodal MRI modalities can further allow assessment of white matter microstructure and functional connectivity, potentially correlating with receptor activity and downstream signaling alterations (83, 84). With PET imaging, researchers can employ radioligands targeting amyloid-*β*, tau, or dopamine transporters to directly quantify pathological protein burden and dopaminergic terminal loss. This approach can be extended to receptor expression studies once suitable VDR/RXR ligands are developed. (85, 86–88) By integrating PET findings with MRI-derived structural and functional data, researchers can assess whether RXR activation enhances VDR function *in vivo*, providing insights into the neuroprotective interplay between these receptors. This multimodal imaging approach could be highly imperative for validating therapeutic strategies involving vitamin D₃ and vitamin A co-supplementation in neurodegenerative disease contexts. Importantly, these clinical imaging modalities have direct preclinical analogs, enabling a seamless translational pipeline from bench to bedside. Techniques such as FLAIR, DTI, and fMRI can be applied in both animal models and humans, ensuring methodological continuity from mechanistic approaches to clinical validation.

## Limitations

Despite the strong mechanistic rationale and supportive preclinical evidence underlying the proposed VDR–RXR synergy hypothesis, several important limitations must be acknowledged.

First, the framework is primarily hypothesis-driven and mechanistic, relying heavily on preclinical models, receptor biology, and molecular signaling logic rather than robust human clinical data. While animal models and *in vitro* systems are indispensable for mechanistic discovery, they do not fully recapitulate the biological, genetic, and environmental complexity of human neurodegenerative diseases. Translational failure is common in neurodegeneration research, and effects observed in rodent models frequently do not reproduce in clinical trials. Therefore, the proposed synergy between vitamin D₃ and vitamin A, although biologically plausible, remains speculative until validated in well-designed human studies.

Second, VDR–RXR signaling is not a linear or isolated pathway. Nuclear receptor biology is inherently complex and context-dependent. Transcriptional outcomes are shaped not only by ligand availability but also by chromatin accessibility, epigenetic state, coactivator/corepressor recruitment, receptor phosphorylation, metabolic status, and cellular redox environment. This means that RXR activation does not guarantee enhanced VDR transcriptional output in all biological contexts. In some conditions, RXR may preferentially dimerize with other nuclear receptors (e.g., PPARs, LXRs, FXR), potentially diverting signaling away from VDR pathways. This receptor competition introduces biological uncertainty into the assumption that RXR activation will consistently potentiate VDR-mediated neuroprotection.

Third, the hypothesis assumes that vitamin A availability is a limiting factor in VDR–RXR signaling, yet this may not universally hold true. Endogenous dietary intake, retinoid metabolism, and brain retinoid homeostasis may already provide sufficient RXR activation in many individuals. In such contexts, additional vitamin A supplementation may offer minimal benefit or no added biological effect. This raises the possibility that combined therapy may only be beneficial in specific subpopulations (e.g., individuals with vitamin A deficiency, altered retinoid metabolism, or impaired RXR signaling), limiting the generalizability of the approach.

Fourth, there are significant safety and dosing concerns, particularly regarding vitamin A. Unlike vitamin D₃, vitamin A has a narrow therapeutic window and well-documented toxicity (hypervitaminosis A), including hepatotoxicity, neuropsychiatric symptoms, and teratogenic effects. Chronic co-supplementation strategies would therefore require extremely careful dose optimization, monitoring, and risk stratification. This creates a translational barrier, as long-term combination therapy may pose clinical risks that outweigh theoretical neuroprotective benefits, especially in elderly or medically vulnerable populations.

Fifth, the current framework lacks direct clinical biomarkers of VDR–RXR activation. Neuroimaging markers such as FLAIR, DTI, and fMRI are indirect downstream readouts of neuroprotection rather than direct measures of receptor signaling. Without specific molecular imaging ligands or validated peripheral biomarkers for VDR–RXR activity, it remains difficult to causally link observed clinical or imaging improvements to receptor heterodimerization mechanisms. This limits mechanistic interpretability in clinical settings and complicates hypothesis testing.

Sixth, neurodegenerative diseases such as Alzheimer’s and Parkinson’s disease are multifactorial systems-level disorders, involving protein aggregation, synaptic dysfunction, immune dysregulation, vascular pathology, mitochondrial failure, and network-level disconnection. Targeting a single receptor axis—even a central regulatory one like VDR–RXR—may be biologically insufficient to meaningfully modify disease trajectories. At best, VDR–RXR modulation is likely to function as an adjunctive strategy rather than a disease-modifying monotherapy.

Finally, the hypothesis does not fully resolve the issue of causality versus association. Observed improvements with combined vitamin treatment in preclinical models may reflect parallel antioxidant, anti-inflammatory, or metabolic effects rather than true receptor-level synergy. Without receptor-specific knockouts, conditional genetic models, and receptor-selective ligands, it remains difficult to definitively attribute neuroprotection to VDR–RXR heterodimerization rather than non-specific pleiotropic vitamin effects.

In summary, while the proposed VDR–RXR combinatorial framework is biologically coherent and mechanistically attractive, it is constrained by translational uncertainty, receptor signaling complexity, safety limitations, biomarker gaps, and disease-level heterogeneity. These limitations do not invalidate the hypothesis, but they clearly position it as a theoretical and exploratory model that requires rigorous molecular validation, receptor-specific mechanistic studies, dose-safety optimization, and carefully controlled clinical trials before it can be considered a viable therapeutic strategy for neurodegenerative diseases.

## Conclusion

Research into neurodegenerative diseases, such as AD and PD, confronts the formidable challenge of progressive neuronal loss. A promising avenue for future investigation lies in elucidating the structural dynamics and mechanistic interplay underlying VDR-RXR heterodimer formation. While preclinical evidence suggests neuroprotective effects of this complex in AD/PD models, outcomes must be weighed against potential risks, including vitamin A toxicity and reports of vitamin D potentially accelerating AD progression in some contexts. By employing complementary experimental approaches to rigorously test this hypothesis, we aim to clarify its therapeutic potential for AD and PD. A multidisciplinary approach is highly needed to validate its efficacy and safety.

## Data Availability

The original contributions presented in the study are included in the article/supplementary material, further inquiries can be directed to the corresponding author/s.

## References

[1] Agnello L Ciaccio M. Neurodegenerative diseases: from molecular basis to therapy. Int J Mol Sci. (2022) 23:12854. doi: 10.3390/ijms232112854, 36361643 PMC9654859

[2] Nowowiejska J Baran A Flisiak I. Psoriasis and neurodegenerative diseases-a review. Front Mol Neurosci. (2022) 15:917751. doi: 10.3389/fnmol.2022.917751, 36226313 PMC9549431

[3] Tolosa E Garrido A Scholz SW Poewe W. Challenges in the diagnosis of Parkinson's disease. Lancet Neurol. (2021) 20:385–97. doi: 10.1016/S1474-4422(21)00030-2, 33894193 PMC8185633

[4] Scheltens P De Strooper B Kivipelto M Holstege H Chételat G Teunissen CE . Alzheimer's disease. Lancet. (2021) 397:1577–90. doi: 10.1016/S0140-6736(20)32205-4 33667416 PMC8354300

[5] Leng F Edison P. Neuroinflammation and microglial activation in Alzheimer disease: where do we go from here? Nat Rev Neurol. (2021) 17:157–72. doi: 10.1038/s41582-020-00435-y, 33318676

[6] Wilson DM 3rd Cookson MR Van Den Bosch L Zetterberg H Holtzman DM Dewachter I . Hallmarks of neurodegenerative diseases. Cell. (2023) 186:693–714. doi: 10.1016/j.cell.2022.12.032 36803602

[7] El Ouaamari Y Van den Bos J Willekens B Cools N Wens I. Neurotrophic factors as regenerative therapy for neurodegenerative diseases: current status, challenges and future perspectives. Int J Mol Sci. (2023) 24:3866. doi: 10.3390/ijms24043866, 36835277 PMC9968045

[8] Ishola AO Laoye BJ Oyeleke DE Bankole OO Sirjao MU Cobham AE . Vitamin D3 receptor activation rescued corticostriatal neural activity and improved motor- cognitive function in −D2R parkinsonian mice model. J Biomed Sci Eng. (2015) 8:601–15.

[9] Bankole OO Laoye BJ Sirjao MU Ishola AO Oyeleke DE Balogun WG . Vitamin D3 receptor activation rescued corticostriatal neural activity and improved motor function in –D2R tardive dyskinesia mice model. J Biomed Sci Eng. (2015) 8:520–30. doi: 10.4236/jbise.2015.88049

[10] Gáll Z Székely O. Role of vitamin D in cognitive dysfunction: new molecular concepts and discrepancies between animal and human findings. Nutrients. (2021) 13:3672. doi: 10.3390/nu13113672, 34835929 PMC8620681

[11] Patel P Shah J. Vitamin D3 supplementation ameliorates cognitive impairment and alters neurodegenerative and inflammatory markers in scopolamine induced rat model. Metab Brain Dis. (2022) 37:2653–67. doi: 10.1007/s11011-022-01086-2, 36156759

[12] Manjari SKV Maity S Poornima R Yau SY Vaishali K Stellwagen D . Restorative action of vitamin D3 on motor dysfunction through enhancement of Neurotrophins and antioxidant expression in the striatum. Neuroscience. (2022) 492:67–81. doi: 10.1016/j.neuroscience.2022.03.039, 35413386

[13] Lin MT Beal MF. Mitochondrial dysfunction and oxidative stress in neurodegenerative diseases. Nature. (2006) 443:787–95. doi: 10.1038/nature05292, 17051205

[14] Johnson J Mercado-Ayon E Mercado-Ayon Y Dong YN Halawani S Ngaba L . Mitochondrial dysfunction in the development and progression of neurodegenerative diseases. Arch Biochem Biophys. (2021) 702:108698. doi: 10.1016/j.abb.2020.108698, 33259796

[15] Teleanu DM Niculescu AG Lungu II Radu CI Vladâcenco O Roza E . An overview of oxidative stress, Neuroinflammation, and neurodegenerative diseases. Int J Mol Sci. (2022) 23:5938. doi: 10.3390/ijms23115938, 35682615 PMC9180653

[16] Azzam AY Ghozy S Azab MA. Vitamin D and its' role in Parkinson's disease patients with SARS-CoV-2 infection. A review article. Interdiscip Neurosurg. (2022) 27:101441. doi: 10.1016/j.inat.2021.101441, 34868885 PMC8627384

[17] Ocanha Xavier JP Xavier JCC Jr da Silva MG Marques MEA. Vitamin D receptor and retinoid X receptor alpha in melanocytic benign lesions and melanoma. Am J Dermatopathol. (2023) 45:619–25. doi: 10.1097/DAD.0000000000002507 37506276

[18] Gezen-Ak D Dursun E. Vitamin D, a secosteroid hormone and its multifunctional receptor, vitamin D receptor, in Alzheimer's type neurodegeneration. J Alzheimer's Dis. (2023) 95:1273–99. doi: 10.3233/JAD-230214 37661883

[19] Lai RH Hsu CC Yu BH Lo YR Hsu YY Chen MH . Vitamin D supplementation worsens Alzheimer's progression: animal model and human cohort studies. Aging Cell. (2022) 21:e13670. doi: 10.1111/acel.13670, 35822270 PMC9381901

[20] Lasoń W Jantas D Leśkiewicz M Regulska M Basta-Kaim A. The vitamin D receptor as a potential target for the treatment of age-related neurodegenerative diseases such as Alzheimer's and Parkinson's diseases: a narrative review. Cells. (2023) 12:660. doi: 10.3390/cells12040660, 36831327 PMC9954016

[21] Umar MS Ibrahim BM. Vitamin a and vitamin D3 protect the visual apparatus during the development of dopamine-2 receptor knockout mouse model of parkinsonism. J Complement Integr Med. (2023) 20:577–89. doi: 10.1515/jcim-2023-0053 37311120

[22] Carlberg C. Genomic signaling of vitamin D. Steroids. (2023) 198:109271. doi: 10.1016/j.steroids.2023.109271 37442517

[23] Salhab A Amer J Yinying L Safadi R. 25(OH) D3 alleviate liver NK cytotoxicity in acute but not in chronic fibrosis model of BALB/c mice due to modulations in vitamin D receptor. BMC Gastroenterol. (2020) 20:102. doi: 10.1186/s12876-020-01248-5, 32276660 PMC7149903

[24] Chen L Yang R Qiao W Zhang W Chen J Mao L . 1,25-Dihydroxyvitamin D exerts an antiaging role by activation of Nrf2-antioxidant signaling and inactivation of p16/p53- senescence signaling. Aging Cell. (2019) 18:e12951. doi: 10.1111/acel.12951, 30907059 PMC6516172

[25] Cui X Eyles DW. Vitamin D and the central nervous system: causative and preventative mechanisms in brain disorders. Nutrients. (2022) 14:4353. doi: 10.3390/nu14204353, 36297037 PMC9610817

[26] Anwar MJ Alenezi SK Alhowail AH. Molecular insights into the pathogenic impact of vitamin D deficiency in neurological disorders. Biomed Pharmacother. (2023) 162:114718. doi: 10.1016/j.biopha.2023.114718, 37084561

[27] Campbell FC Xu H El-Tanani M Crowe P Bingham V. The yin and yang of vitamin D receptor (VDR) signaling in neoplastic progression: operational networks and tissue- specific growth control. Biochem Pharmacol. (2010) 79:1–9. doi: 10.1016/j.bcp.2009.09.005, 19737544 PMC2824849

[28] Ladumor Y Seong BKA Hallett R Valencia-Sama I Adderley T Wang Y . Vitamin D receptor activation attenuates hippo pathway effectors and cell survival in metastatic neuroblastoma. Mol Cancer Res. (2022) 20:895–908. doi: 10.1158/1541-7786.MCR-21-0425 35190818 PMC9177824

[29] Tripathi AK Mishra SK. A review article on neuroprotective, immunomodulatory, and anti-inflammatory role of vitamin-D3 in elderly COVID-19 patients. Egypt J Neurol Psychiatr Neurosurg. (2023) 59:18. doi: 10.1186/s41983-023-00611-z, 36776226 PMC9901404

[30] Djais AI Oktawati S Thahir H Hatta M Sukmana BI Dewi N . Analysis of VDR gene polymorphism in Beta thalassemia major (Beta thalassemia major/vitamin D/calcium/825T/T vitamin D receptor gene). Syst Rev Pharm. (2020) 11:515–22. doi: 10.31838/srp.2020.4.39

[31] Singh RK Turner R Kim K Sivagnanalingam U Moore RG. Targeting vitamin D receptor (VDR)/immune checkpoint inhibitor receptor ligand PD-L1 axis for immunotherapy of ovarian cancer. Gynecol Oncol. (2018) 149:48. doi: 10.1016/j.ygyno.2018.04.103

[32] Heublein S Mayr D Meindl A Kircher A Jeschke U Ditsch N. Vitamin D receptor, retinoid X receptor and peroxisome proliferator-activated receptor γ are overexpressed in BRCA1 mutated breast cancer and predict prognosis. J Exp Clin Cancer Res. (2017) 36:57. doi: 10.1186/s13046-017-0517-1, 28427429 PMC5399435

[33] Pérez E Bourguet W Gronemeyer H de Lera AR. Modulation of RXR function through ligand design. Biochim Biophys Acta. (2012) 1821:57–69. doi: 10.1016/j.bbalip.2011.04.003, 21515403

[34] Haussler MR Whitfield GK Kaneko I Haussler CA Hsieh D Hsieh JC . Molecular mechanisms of vitamin D action. Calcif Tissue Int. (2013) 92:77–98. doi: 10.1007/s00223-012-9619-0 22782502

[35] Martorell S Hueso L Gonzalez-Navarro H Collado A Sanz MJ Piqueras L. Vitamin D receptor activation reduces angiotensin-II-induced dissecting abdominal aortic aneurysm in Apolipoprotein E-knockout mice. Arterioscler Thromb Vasc Biol. (2016) 36:1587–97. doi: 10.1161/ATVBAHA.116.307530, 27283745

[36] Amini Y Hamid YH Cheung PCK. Vitamins and their roles in neurodegenerative diseases. Front Nutr. (2023)

[37] Ricci C. Neurodegenerative disease: from molecular basis to therapy. Int J Mol Sci. (2024) 25:967. doi: 10.3390/ijms25020967, 38256040 PMC10815646

[38] Aggeletopoulou I Thomopoulos K Mouzaki A Triantos C. Vitamin D-VDR novel anti- inflammatory molecules-new insights into their effects on liver diseases. Int J Mol Sci. (2022) 23:8465. doi: 10.3390/ijms23158465, 35955597 PMC9369388

[39] Li A Yi B Han H Yang S Hu Z Zheng L . Vitamin D-VDR (vitamin D receptor) regulates defective autophagy in renal tubular epithelial cell in streptozotocin-induced diabetic mice via the AMPK pathway. Autophagy. (2022) 18:877–90. doi: 10.1080/15548627.2021.1962681, 34432556 PMC9037529

[40] Christakos S Dhawan P Verstuyf A Verlinden L Carmeliet G. Vitamin D: metabolism, molecular mechanism of action, and pleiotropic effects. Physiol Rev. (2016) 96:365–408. doi: 10.1152/physrev.00014.2015, 26681795 PMC4839493

[41] Rehó B Fadel L Brazda P Benziane A Hegedüs É Sen P . Agonist-controlled competition of RAR and VDR nuclear receptors for heterodimerization with RXR is manifested in their DNA binding. J Biol Chem. (2023) 299:102896. doi: 10.1016/j.jbc.2023.102896, 36639026 PMC9943875

[42] Mazzetti S Barichella M Giampietro F Giana A Calogero AM Amadeo A . Astrocytes expressing vitamin D-activating enzyme identify Parkinson's disease. CNS Neurosci Ther. (2022) 28:703–13. doi: 10.1111/cns.13801, 35166042 PMC8981451

[43] Ye X Zhou Q Ren P Xiang W Xiao L. The synaptic and circuit functions of vitamin D in neurodevelopment disorders. Neuropsychiatr Dis Treat. (2023) 19:1515–30. doi: 10.2147/NDT.S407731, 37424961 PMC10327924

[44] Eyles DW. Vitamin D: brain and behavior. JBMR Plus. (2020) 5:e10419. doi: 10.1002/jbm4.10419, 33553986 PMC7839822

[45] Eyles DW Burne TH McGrath JJ. Vitamin D in fetal brain development. Semin Cell Dev Biol. (2013) 24:626–36. 10.1016/j.semcdb.2011.05.004 21664981

[46] Rochel N Wurtz JM Mitschler A Klaholz B Moras D. The crystal structure of the nuclear receptor for vitamin D bound to its natural ligand. Mol Cell. (2000) 5:173–9. doi: 10.1016/S1097-2765(00)80413-X, 10678179

[47] Dursun E Gezen-Ak D Yilmazer S. A new mechanism for amyloid-β induction of iNOS: vitamin D-VDR pathway disruption. J Alzheimer's Dis. (2013) 36:459–74. doi: 10.3233/JAD-130416, 23624519

[48] Umar SM Usman Y Atuadu V Shehu K Oyem JC Badamasi IM. Vitamin D3 and vitamin a synergistically prevent visuospatial memory impairment in mice model of the dopaminergic system disorder. J Nutr Biochem. (2026) 148:110145. doi: 10.1016/j.jnutbio.2025.110145 41093077

[49] Mizwicki MT Norman AW. The vitamin D sterol–vitamin D receptor ensemble model offers unique insights into both genomic and rapid-response signaling. Sci Signal. (2009) 16:275re4. doi: 10.1126/scisignal.275re4, 19531804

[50] Haussler MR Whitfield GK Haussler CA Hsieh JC Thompson PD Selznick SH . The nuclear vitamin D receptor: biological and molecular regulatory properties revealed. J Bone Miner Res. (1998) 13:325–49. doi: 10.1359/jbmr.1998.13.3.325, 9525333

[51] Guo CH Cao T Zheng LT Waddington JL Zhen XC. Development and characterization of an inducible dicer conditional knockout mouse model of Parkinson's disease: validation of the antiparkinsonian effects of a sigma-1 receptor agonist and dihydromyricetin. Acta Pharmacol Sin. (2020) 41:499–507. doi: 10.1038/s41401-020-0379-5, 32112040 PMC7468551

[52] Ham HJ Yeo IJ Jeon SH Lim JH Yoo SS Son DJ . Botulinum toxin a ameliorates Neuroinflammation in the MPTP and 6-OHDA-induced Parkinson's disease models. Biomol Ther. (2022) 30:90–7. doi: 10.4062/biomolther.2021.077, 34078752 PMC8724835

[53] Jackson-Lewis V Przedborski S. Protocol for the MPTP mouse model of Parkinson's disease. Nat Protoc. (2007) 2:141–51. doi: 10.1038/nprot.2006.342 17401348

[54] Sirajo MU Oyem JC Badamasi MI. Supplementation with vitamins D3 and a mitigates Parkinsonism in a haloperidol mice model. J Chem Neuroanat. (2023) 135:102366. doi: 10.1016/j.jchemneu.2023.102366 38040269

[55] Sirajo MU Owolabi LF Abubakar M Olakunle IA Tela A Shehu K . Ameliorative effect of vitamin C and UV-B rays on Nigrostriatal and Corticostriatal neural degeneration in haloperidol induced Parkinsonism in Wistar rats. Nig J Neurosci. (2019) 10:61–70.

[56] Götz J Ittner LM. Animal models of Alzheimer's disease and frontotemporal dementia. Nat Rev Neurosci. (2008) 9:532–44. doi: 10.1038/nrn2420 18568014

[57] Jucker M Walker LC. Pathogenic protein seeding in Alzheimer disease and other neurodegenerative disorders. Ann Neurol. (2011) 70:532–40. doi: 10.1002/ana.22615, 22028219 PMC3203752

[58] Le Bihan D Mangin JF Poupon C Clark CA Pappata S Molko N . Diffusion tensor imaging: concepts and applications. J Magn Reson Imaging. (2001) 13:534–46. doi: 10.1002/jmri.1076, 11276097

[59] Logothetis NK. What we can do and what we cannot do with fMRI. Nature. (2008) 453:869–78. doi: 10.1038/nature06976 18548064

[60] Thompson PM Hayashi KM de Zubicaray GI Janke AL Rose SE Semple J . Mapping hippocampal and ventricular change in Alzheimer disease. NeuroImage. (2004) 22:1754–66. doi: 10.1016/j.neuroimage.2004.03.040 15275931

[61] Teipel SJ Bokde ALW Meindl T Amaro E Soldner J Reiser MF . White matter microstructure underlying default mode network connectivity in the human brain. NeuroImage. (2010) 49:2021–32. doi: 10.1016/j.neuroimage.2009.10.067 19878723

[62] Damoiseaux JS Rombouts SA Barkhof F Scheltens P Stam CJ Smith SM . Consistent resting-state networks across healthy subjects. Proc Natl Acad Sci USA. (2006) 103:13848–53. doi: 10.1073/pnas.0601417103, 16945915 PMC1564249

[63] Edison P Archer HA Hinz R Hammers A Pavese N Tai YF . Amyloid, hypometabolism, and cognition in Alzheimer disease: an [11C]PIB and [18F]FDG PET study. Neurology. (2007) 68:501–8. doi: 10.1212/01.wnl.0000244749.20056.d4 17065593

[64] Villemagne VL Pike KE Darby D Maruff P Savage G Ng S . Abeta deposits in older non-demented individuals with cognitive decline are indicative of preclinical Alzheimer's disease. Neuropsychologia. (2008) 46:1688–97. doi: 10.1016/j.neuropsychologia.2008.02.008, 18343463

[65] Coughlin JM Wang Y Ambinder EB Ward RE Minn I Vranesic M . In vivo markers of VDR and RXR expression: progress and perspectives for PET radioligand development. Mol Imaging Biol. (2021) 23:453–64.

[66] Annweiler C Llewellyn DJ Beauchet O. Low serum vitamin D concentrations in Alzheimer's disease: a systematic review and meta-analysis. J Alzheimer's Dis. (2013) 33:659–74. doi: 10.3233/JAD-2012-121432, 23042216

[67] Fleming SM Ekhator OR Ghisays V. Assessment of sensorimotor function in mouse models of Parkinson's disease. J Vis Exp. (2013) 76:e50303. doi: 10.3791/50303 PMC3727502 23851663

[68] Sirajo MU Murtala K Oyem JC Olakunle IA Owolabi LF. Motor function test protocol for parkinsonian triad in rodent model of Parkinson's disease. J Neuro Behav Sci. (2022) 9 ISSN:2149-1909:1–6. doi: 10.4103/jnbs.jnbs_1_22

[69] Deacon RM. Measuring the strength of mice. J Vis Exp. (2013) 76:e2610. doi: 10.3791/2610, 23770643 PMC3725666

[70] Vorhees CV Williams MT. Morris water maze: procedures for assessing spatial and related forms of learning and memory. Nat Protoc. (2006) 1:848–58. doi: 10.1038/nprot.2006.116, 17406317 PMC2895266

[71] Kraeuter AK Guest PC Sarnyai Z. The Y-maze for assessment of spatial working and reference memory in mice. Methods Mol Biol. (2019) 1916:105–11. doi: 10.1007/978-1-4939-8994-2_10 30535688

[72] Ennaceur A Delacour J. A new one-trial test for neurobiological studies of memory in rats. 1: behavioral data. Behav Brain Res. (1988) 31:47–59. doi: 10.1016/0166-4328(88)90157-X 3228475

[73] Guskjolen AJ Josselyn SA Frankland PW. Fluoxetine impairs performance in the novel object recognition task by increasing exploration. Behav Brain Res. (2016) 296:15–25. doi: 10.1016/j.bbr.2015.08.027 26315459

[74] Sharma S Rakoczy S Brown-Borg H. Assessment of spatial memory in mice. Life Sci. (2010) 87:521–36. doi: 10.1016/j.lfs.2010.09.003, 20837032 PMC6457258

[75] LeDoux JE. Emotion circuits in the brain. Annu Rev Neurosci. (2000) 23:155–84. doi: 10.1146/annurev.neuro.23.1.155 10845062

[76] Eichenbaum H. Hippocampus: cognitive processes and neural representations that underlie declarative memory. Neuron. (2004) 44:109–20. doi: 10.1016/j.neuron.2004.08.028 15450164

[77] Mahmood T Yang PC. Western blot: technique, theory, and trouble shooting. N Am J Med Sci. (2012) 4:429–34. doi: 10.4103/1947-2714.100998. 23050259, 23050259 PMC3456489

[78] Arya M Shergill IS Williamson M Gommersall L Arya N Patel HR. Basic principles of real-time quantitative PCR. Expert Rev Mol Diagn. (2005) 5:209–19. doi: 10.1586/14737159.5.2.209 15833050

[79] Badamasi IM Tajudeen A Owolabi SD Ojeahere MI Yusuf AA Sirajo MU . "waist- height ratio highlights detrimental risk for olanzapine associated weight gain earlier than body mass index" international journal of adolescent. Medicine Health. (2024) 36:579–85. doi: 10.1515/ijamh-2024-0099 39432346

[80] Zella JB Meyer MB Nerenz RD Lee SM Martowicz ML Pike JW. Multifunctional enhancer regulation by the vitamin D receptor in a ligand -dependent manner. Mol Endocrinol. (2010) 24:128–47. doi: 10.1210/me.2009-0274, 19897601 PMC2802900

[81] Buoso E Lanni C Molteni E Rousset F Costa A Schettini G . Opposite roles of the alpha7 nicotinic receptor in neuroprotection and neurodegeneration. Biochem Pharmacol. (2011) 82:278–86. doi: 10.1016/j.bcp.2011.05.003 21600888

[82] Jack CR Jr Knopman DS Jagust WJ Shaw LM Aisen PS Weiner MW . Hypothetical model of dynamic biomarkers of the Alzheimer's pathological cascade. Lancet Neurol. (2010) 9:119–28. doi: 10.1016/S1474-4422(09)70299-6, 20083042 PMC2819840

[83] Le Bihan D. Looking into the functional architecture of the brain with diffusion MRI. Nat Rev Neurosci. (2003) 4:469–80. doi: 10.1038/nrn1119, 12778119

[84] Fox MD Raichle ME. Spontaneous fluctuations in brain activity observed with functional magnetic resonance imaging. Nat Rev Neurosci. (2007) 8:700–11. doi: 10.1038/nrn2201 17704812

[85] Villemagne VL Fodero-Tavoletti MT Masters CL Rowe CC. Tau imaging: early progress and future directions. Lancet Neurol. (2015) 14:114–24. doi: 10.1016/S1474-4422(14)70252-2 25496902

[86] Pike VW. PET radiotracers: crossing the blood –brain barrier and surviving metabolism. Trends Pharmacol Sci. (2009) 30:431–40. doi: 10.1016/j.tips.2009.06.005, 19616318 PMC2805092

[87] Elsworth JD. Parkinson's disease treatment: past, present, and future. J Neural Transm. (2020) 127:785–91. doi: 10.1007/s00702-020-02167-1, 32172471 PMC8330829

[88] Nishikawa J Kitaura M Matsumoto M Imagawa M Nishihara T. Difference and similarity of DNA sequence recognized by VDR homodimer and VDR/RXR heterodimer. Nucleic Acids Res. (1994) 22:2902–7. doi: 10.1093/nar/22.15.2902, 8065900 PMC310253

